# Bioproduct Potential of Outdoor Cultures of *Tolypothrix* sp.: Effect of Carbon Dioxide and Metal-Rich Wastewater

**DOI:** 10.3389/fbioe.2020.00051

**Published:** 2020-02-11

**Authors:** Chinnathambi Velu, Samuel Cirés, Diane L. Brinkman, Kirsten Heimann

**Affiliations:** ^1^North Queensland Algal Identification Facility, Aquaculture, College of Science and Engineering, James Cook University, Townsville, QLD, Australia; ^2^Departamento de Biología, Universidad Autónoma de Madrid, Madrid, Spain; ^3^Australian Institute of Marine Science (AIMS), Townsville, QLD, Australia; ^4^Centre for Marine Bioproducts Development (CMBD), College of Medicine and Public Health, Flinders University, Bedford Park, SA, Australia

**Keywords:** biofertilizer, bioremediation, metals, biorefinery, coal-fired power, phycobiliproteins, nitrogen-fixing cyanobacteria, economics

## Abstract

Rising CO_2_ levels, associated climatic instability, freshwater scarcity and diminishing arable land exacerbate the challenge to maintain food security for the fast growing human population. Although coal-fired power plants generate large amounts of CO_2_ emissions and wastewater, containing environmentally unsafe concentrations of metals, they ensure energy security. Nitrogen (N_2_)-fixation by cyanobacteria eliminate nitrogen fertilization costs, making them promising candidates for remediation of waste CO_2_ and metals from macronutrient-poor ash dam water and the biomass is suitable for phycocyanin and biofertilizer product development. Here, the effects of CO_2_ and metal mixtures on growth, bioproduct and metal removal potential were investigated for the self-flocculating, N_2_-fixing freshwater cyanobacterium *Tolypothrix* sp. *Tolypothrix* sp. was grown outdoors in simulated ash dam wastewater (SADW) in 500 L vertical bag suspension cultures and as biofilms in modified algal-turf scrubbers. The cultivation systems were aerated with air containing either 15% CO_2_ (v/v) or not. CO_2_-fertilization resulted in ∼1.25- and 1.45-fold higher biomass productivities and ∼40 and 27% increased phycocyanin and phycoerythrin contents for biofilm and suspension cultures, respectively. CO_2_ had no effect on removal of Al, As, Cu, Fe, Sr, and Zn, while Mo removal increased by 37% in both systems. In contrast, Ni removal was reduced in biofilm systems, while Se removal increased by 73% in suspension cultures. Based on biomass yields and biochemical data obtained, net present value (NPV) and sensitivities analyses used four bioproduct scenarios: (1) phycocyanin sole product, (2) biofertilizer sole product, (3) 50% phycocyanin and 50% biofertilizer, and (4) 100% phycocyanin and 100% biofertilizer (residual biomass) for power station co-located and not co-located 10 ha facilities over a 20-year period. Economic feasibility for the production of food-grade phycocyanin either as a sole product or with co-production of biofertilizer was demonstrated for CO_2_-enriched vertical and raceway suspension cultures raised without nitrogen-fertilization and co-location with power stations significantly increased profit margins.

## Graphical Abstract

Framework for an integrated bio-economic model for co-locating cyanobacterial cultivation with coal-fired power station based on a biorefinery approach.

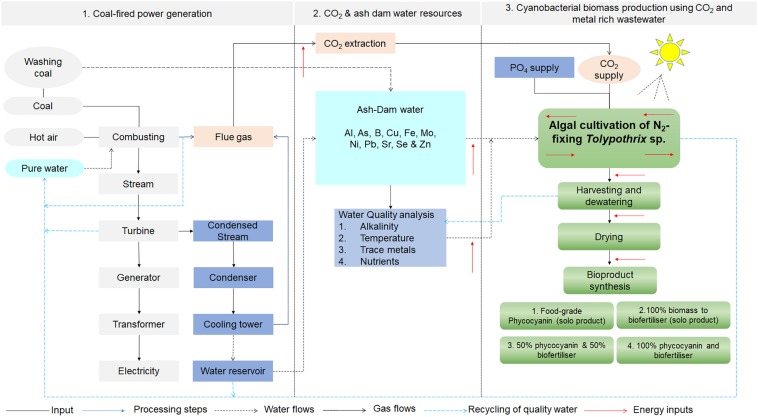

## Introduction

Anthropogenic emissions of carbon dioxide (CO_2_) account for 68% of total emissions (Ho et al., 2011), posing a threat to the global climatic equilibrium. At present, coal-powered electricity generation is still required in Australia and globally to meet energy requirements and – security (Stock, 2014). Flue gas from coal-fired power plants contain 10–15% CO_2_ (v/v) and generate wastewater enriched with heavy metals (Artanto et al., 2014). Biological fixation of CO_2_ and absorption of nutrients/metals from wastewaters by photosynthetic organisms such as microalgae and cyanobacteria is gaining industrial interest, as the biomass produced can yield a variety of high- and low-value renewable products (Wang et al., 2008). Wastewater generated at coal-fired power plants (ash dam water) cannot be discharged due to its potential toxicity and is therefore stored in ash dams (Roberts et al., 2015; Velu et al., 2019). The ash dam water contains metals, many of which serve as micronutrients important for plant growth, but macro-nutrients, such as nitrogen and phosphate, are lacking (Saunders et al., 2012). As the cultivation of eukaryotic photosynthetic organisms requires nitrogen and phosphate for growth, ash dam water needs to be supplemented with these macro-nutrients, increasing the cost of bioremediation and biomass production (Velu et al., 2019). In contrast, the cultivation of diazotrophic cyanobacteria does not require nitrogen fertilization, as these organisms can fix atmospheric nitrogen (N_2_), making them an ideal choice for bioremediation of metals from nitrogen-limited wastewaters (Markou and Georgakakis, 2011). This is a clear advantage, as globally 85 million tons of nitrogenous fertilizer were used in 2000 for food production. Synthetic nitrogenous fertilizers are projected to not meet the demands of the ever growing human population in the near future (Singh et al., 2016). In addition, the current exploitation of chemically derived fertilizers have been shown to contribute to environmental problems, such as pollution, reduced soil fertility and adverse impacts on the ozone layer (Benemann, 1979; Singh et al., 2016). In an Australian context, agricultural productivity is declining in regions with marginal/leached soils, as microbial consortia, essential for soil fertility, are negatively affected by declining soil carbon contents. Soil fertility cannot be improved by provision of nitrogen without the addition of large amounts of carbon (QLD, 2016). Despite the realization of adverse impacts of synthetic nitrogenous fertilizers, ∼5.3 million tonnes of chemical fertilizers were used on 49.1 million ha agricultural land in Australia between 2014–2015 (ABS, 2015). Thus, there is a pressing need for the sustainable production of innovative fertilizers that are effective, renewable, environmentally friendly, cost-efficient, and improve soil fertility to ensure food security in the future. In this context, fertilizers derived from biological nitrogen fixation and through recycling and re-use of nitrogen contained in various wastewaters offer great potential benefits (Benemann, 1979; Singh et al., 2016).

Nitrogen fixation is carried out by the oxygen-sensitive, iron and molybdenum-containing nitrogenase complex (Abed et al., 2009), resulting in higher iron and molybdenum requirements, elements that are present at elevated concentrations in ash dam wastewater (Saunders et al., 2012). While excessive metal concentrations can retard cyanobacterial growth (Pereira et al., 2011), species such as *Anabaena subcylindrica*, *Aphanocapsa* sp., *Calothrix* sp., *Microcystis* sp., *Oscillatoria salina*, *Plectonema terebrans*, and *Synechococcus* sp. can be used for the treatment of domestic and industrial wastewater (Dubey et al., 2011). Biomineralisation of metals by cyanobacteria occurs via intracellular bioaccumulation and/or passive biosorption, the latter is mediated by the presence of an exopolysaccharide layer (EPS) on the outside of many cyanobacterial species (Pereira et al., 2011). The EPS consists of complex heteropolysaccharides on a glucosamine backbone, providing an accumulation of negative charges that play an essential role in the chelation of metal ions (De Philippis et al., 2011). On a dry weight basis, *Calothrix scopulorum* and *C. marchica* chelated 0.7 and 6.4% of lead (Weckesser et al., 1988; Ruangsomboon et al., 2007). *Nostoc muscorum* chelated 22.5, 11.8, 26.4, and 32% of copper, cobalt, lead and manganese, while *Anabaena subcylindrica* performed much better (81.8. 33.7, 100, and 100%, respectively) (El-Sheekh et al., 2005). The large differences in the biosorption of metals indicates that the choice of species is an important criterion to consider, especially when the reutilization of large volumes of wastewater is an important aspect of the industrial process. As CO_2_-fertilization enhances cyanobacterial growth (Velu et al., 2015, 2019), EPS content will increase simultaneously, which should enhance metal chelation capacity of the cultures and, hence, remediation capacity. The presence of complex metals mixtures in industrial wastewaters may, however, result in competition for the same binding sites, which can result in reduced adsorption efficiencies (Pereira et al., 2011). The diazotrophic filamentous freshwater cyanobacterium, *Tolypothrix* sp. has been used for treatment of domestic and industrial wastewaters and *T. ceytonica* achieved an 86 and 64.4% efficiency for the removal of zinc and total suspended solids (El-Bestawy, 2008).

In addition to the exploitation of environmental services (CO_2_ and metal remediation), the prokaryotic cyanobacteria show additional advantages for biotechnological applications, such as strain-dependent wide environmental tolerances, e.g., marine to freshwater, acid and/or alkaline conditions (Gupta et al., 2013), rapid growth and high photosynthetic activities (Hall et al., 1995). Furthermore, the produced biomass has multiple commercial applications through bioproduct development. In general, potential algal bioproducts include medicinal compounds, food and feed supplements (restricted to CO_2_ enriched grown species without inclusion of metal- or other potentially toxic compound-containing wastewater treatment), pigments (e.g., β-carotene, astaxanthin, fucoxanthin, lutein, phycocyanin, phycoerythrin, the latter two from cyanobacteria), protein, carbohydrate, biofuel and biohydrogen, and biofertilizers (Setta et al., 2017; von Alvensleben and Heimann, 2019). Specific cyanobacterial bioproducts could be protein, mineral and unsaturated fatty acid supplements and the pigments phycocyanin and phycoerythrin from *Arthrospira platensis* or *Limnospira maxima* (formerly *Spirulina platensis* and *S. maxima*), where the protein content of the biomass can reach 74% (Cohen, 1997), which can be extracted through biorefining (Borowitzka, 2013). It might be argued that cyanobacterial biomass produced using metal-rich wastewater is not suitable for high-value phycocyanin product development. Indeed some binding of iron and mercury, the latter not present in ash dam water of coal-fired power plants used in this study, and less efficient binding of some other metals to phycocyanin has been described (Bermejo et al., 2008; Gelagutashvili and Tsakadze, 2013; Bhayani et al., 2016). It is, however, unclear how much binding would occur and how irreversible the binding would be. In addition, affinity of the metals present in ash dam water can be expected traditional metal chelating proteins, such as metallothioneins, and the highly negatively charged EPS, both present in cyanobacterial including *Tolypothrix* sp. biomass, would be more efficient binding sites. In addition, as proposed in this study, phycocyanin extracted from *Tolypothrix* sp. biomass will be purified to upgrade the product to food-grade phycocyanin to obtain a higher sales price, which would further remove any metals bound to phycocyanin. It will nonetheless be essential to analyze the final product for metal contents for quality assurance.

While light, temperature and CO_2_ supplies can be easily controlled at laboratory scale, therefore producing best biomass yields, biomass productivities are typically reduced in large volume suspension-based systems, due to light and carbon limitation, particularly in raceway pond cultivation (Pierobon et al., 2018). Under outdoor large-scale cultivation conditions, improved solar and carbon supplies can be achieved in closed bioreactors, but this adds energy and infrastructure costs, limiting suitability to high-value product development (Pierobon et al., 2018). In contrast, cyanobacterial biofilm reactors are better suited for cost-effective biomass production and are frequently used for wastewater treatment (Hoh et al., 2016). Cyanobacterial biofilm cultivation requires minimal water supplies, gas exchange (CO_2_ absorption and O_2_ venting) is more efficient and harvesting is energy-efficient (Heimann, 2016). Recently developed porous substrate biofilm reactors show efficient light –, carbon – and water utilization and scale-up of this technology is easily possible, making them a promising technology for economical microalgal/microbial biomass production (Pierobon et al., 2018). For example, cyanobacterial biomass productivity was greater in rotating biofilm reactors without aeration or additional CO_2_-supplementation compared to suspension reactors (Gross and Wen, 2014), but the adhesion process for mat establishment is sensitive to shear forces, and species- and substrate-dependent.

Integrated biomass production with wastewater and CO_2_ emission-generating industries has many advantages, i.e., use of non-arable land, non-potable water and provision of trace metals, and CO_2_ to support biomass and bioproduct productivities (Roberts et al., 2015; Moheimani, 2016; Aslam et al., 2019). Nonetheless, economic feasibility still needs to be demonstrated on a case-by-case basis, as outcomes are dependent on the value of the bioproduct(s) and yields. In addition, in the case of ash dam water generated at coal-fired power plants, metal toxicity may occur, reducing yields and application potential of generated bioproducts (Velu et al., 2019). Previous research established that the diazotrophic *Tolypothrix* sp. (isolated from tropical Australia) efficiently self-flocculates, reducing energy requirements for harvesting/dewatering of biomass by 90% (Heimann et al., 2013; Velu et al., 2015). Furthermore, no metal toxicity was observed for *Tolypothrix* sp. biomass production in simulated ash dam water (SADW) and growth performance was independent of nitrogen supply, yet costs for phosphate fertilization are incurred (Velu et al., 2019). The graphical abstract illustrates the integrated production of *Tolypothrix* sp. biomass and potential bioproducts when co-located at a coal-fired power plant.

Therefore, this study used the Australian isolate of *Tolypothrix* sp. to contrast biomass productivities, metal removal capacity and bioproduct potential for biomass cultivated in simulated ash dam water (SADW) between a traditional bubble column reactor and a modified algal turf scrubber with and without CO_2_ supplementation under outdoor conditions. Additionally, the economic feasibility for bioproduct development was estimated, considering four scenarios: (1) production of food-grade phycocyanin as a sole product, (2) biofertilizer as a sole product, (3) use of half the biomass for biofertilizer and food-grade phycocyanin production and (4) biorefining of the high-value phycocyanin with the residue being used as biofertilizer. This study modeled net present value (NPV) and sensitivity analysis for these four scenarios under conditions of co-location with coal-fired power plants and traditional cultivation (not co-located) for a 10 ha plant using suspension bubble columns and raceways for biomass production.

## Materials and Methods

### Culture Collection and Strain Characterization

The diazotrophic, filamentous, freshwater cyanobacterial strain *Tolypothrix* sp. NQAIF319 was isolated and maintained as described in Velu et al. (2015).

### Synthetic Ash Dam Wastewater Preparation

Synthetic ash dam wastewater was prepared as described in Velu et al. (2019) based on concentrations obtained for ash dam water of a Queensland coal-fired power plant, Australia (Saunders et al., 2012).

### Outdoor Cultivation Set Up, Growth Estimation and Biochemical Profiling

Outdoor cultivation of *Tolypothrix* sp. NQAIF319 occurred in SADW in four meso-scale open bioreactor prototypes: (1) two algal turf scrubbers (ATS) of 2.2 m^2^ each (2.2 m long × 1 m wide) (Supplementary Figure S1 and Supplementary Table S1); (2) two suspension vertical bags of 500 L each with a 0.3 m^2^ area footprint, designed and assembled at James Cook University Australia (Supplementary Figure S2 and Supplementary Table S2). The cultivation area was shaded with a UV-shade cloth (Coolaroo, 3.66 m wide, 60% shading) to control photon flux density between 500 and 900 μmol m^–2^ s^–1^ at the Freshwater Compound at James Cook University, Townsville, Australia (19.33 S, 146.76 E). The plastic trays of the ATSs were lined with polystyrene for attachment of *Tolypothrix* sp. and had a slope of 7%. Water flow conditions were continuous at 66 L min^–1^ delivered from a sump with a 500 L fill volume beneath the ATS. The 500 L vertical bubbled suspension culture systems were constructed from PVC bag material contained in a wire cage with a plastic keeled footing of 0.3 m^2^. A tap was fitted to the bottom corner of the vertical bag for harvesting, while aeration was provided by a suspended diffuser delivering 0.05 L air L^–1^ min^–1^ from the main compressor.

The two ATS tanks and two suspension vertical bags were filled with 500 L SADW. Outdoor culture inoculi (starter cultures) were grown in 20 L polycarbonate carboys in BG11 medium without nitrogen [BG11(-N)] at 28°C and a photon flux density of 100 μmol m^–2^ s^–1^ until cultures reached stationary phase. For inoculation of the vertical bag system, the starter cultures were centrifuged (8,000 × *g*, 20 min; Beckman Avanti^®^ J-26XP, Australia) and an adequate volume of the cell pellet was resuspended into 500 L of fresh SADW medium to reach an initial biomass concentration of 0.1 g dry weight L^–1^. The ATS were inoculated by spreading the centrifuged *Tolypothrix* sp. biomass to an initial concentration of 5 g DW m^–2^ without water flow. After an overnight attachment period, water was supplied to the top of the system via a baffle.

A total of 16-day growth experiments were simultaneously performed in both systems in two consecutive runs, during September 2016 (run 1) and October 2016 (run 2). In both runs, one set of each cultivation system was supplemented with CO_2_-enriched air (15% v/v), while the other sets were supplied with air at atmospheric CO_2_ levels (non-CO_2_ controls). CO_2_-enriched air and air were baffled in both cultivation systems. Gases were 99.9% pure, ISO certified and supplied by BOC, a member of the Linde Group, Townsville, Australia. Temperature and pH were monitored twice daily (WP-81, TPS Instruments, Australia) and irradiance was measured at the time of sampling with a LI-250A photosynthetic active radiation (PAR) light probe (LiCor, Biosciences, United States). The incident sun light at sample time varied between 500 and 700 μmol m^–2^ s^–1^. Due to infrastructure and space limitations, replication for each system was carried out sequentially. Thus changes in the outdoor environmental parameters, such as light, temperature, and humidity, the latter especially affecting the biofilm cultivation system, is expected to additionally influence biomass production and metabolic profiles, rendering formal statistical analysis of the data inappropriate.

Samples were collected via a tap for the vertical suspension bag systems and by scraping a 100 cm^–2^ biofilm square (10 × 10 cm) using a silicone rubber cell scraper (IWAKI, Japan) every 4 days. Biomass growth (g DW m^–2^) was determined gravimetrically. Biomass productivities (g DW m^–2^ day^–1^) were calculated as per von Alvensleben et al. (2013), while doubling rate (*k*) and doubling time (T_2_) calculations followed Gour et al. (2014). Water samples for nutrient and metal analyses were collected from the ATS sumps and the supernatant of the centrifuged samples of the vertical suspension culture systems. Culture media phosphate and metal concentrations followed procedures described in detail in Velu et al. (2019), and biomass growth (g DW m^–2^) and productivity (g DW m^–2^ day^–1^) was determined gravimetrically (von Alvensleben et al., 2013) on days 0, 4, 8, 12, and 16. Biomass-specific growth rates (μ_1__–__3_), doubling rate (k) and doubling time (T2) were calculated as per Gour et al. (2014) and von Alvensleben et al. (2013).

Biomass was harvested on day 16 and biomass pellets were freeze-dried (Dynavac model Fd12, Australia) and stored in the dark at −80°C (Sanyo MDF-U33V, Japan) until analyses. Procedures for analyses of pigments (phycocyanin and phycoerythrin), fatty acids (fatty acid methyl esters (FAME), total lipid, alkane/alkene, protein, carbohydrate and carbon, hydrogen, sulphur, phosphorous, potassium (CHNSPK) contents followed Velu et al. (2019).

All chemicals and solvents were obtained from Sigma-Aldrich, Sydney, Australia.

### Net Present Value (NPV) and Sensitivity Analyses

A techno-economic cost assessment was used to evaluate the economic feasibility for production of food-grade phycocyanin and biofertilizer from *Tolypothrix* sp. biomass, assuming either co-location with a coal-fired power plant or traditional cultivation, which is classified here as biomass production solely for bioproduct development without co-location with either an industrial wastewater producer and/or CO_2_ emitter. The following boundaries were set: (1) The algae cultivation plant has a 10 ha cultivation area and employs suspension-based open cultivation systems with an annual biomass production period of 300 days year^–1^. (2) The operating life was set to 20 years. (3) Production data generated in this study in vertical suspension culture systems in SADW and supplied with 15% CO_2_ (v/v) and average biomass productivities achieved for *Nannochloropsis oculata* in large-scale outdoor raceways formed the basis of the analyses. (4) All costs were adjusted to increase by 5% annually, while production sale prices were not adjusted. (5) Food-grade phycocyanin production assumed an extraction/purification efficiency of 67% (Chaiklahan et al., 2018) and a sale price for total phycocyanin of US$ 500 kg^–1^ (Querques et al., 2015), while the sale price for biofertilizer was estimated to be US$ 500 t^–1^.

The NPV was calculated by difference between the present value of cash income and the present value of all cash expenditures following the equation

$$N\,P\,V=\sum_{t=1}^{r}\frac{C_{t}}{{\left(1+r\right)}^{2}}-C_{0}$$

where *C*_*t*_ is the net cash flow during the period t, *C*_0_ is the total initial investment cost, t is the number of time periods (years) and r is the discount rate.

The weighted average costs of capital (WACC) was set to 10% and sensitivity analyses were performed for two scenarios: (1) a reduced biofertilizer price of 25% of the current value (US$ 125 t^–1^) and (2) a reduced sales price for food-grade phycocyanin at 25% of today’s price (US$ 125 kg^–1^).

## Results

### Effect of Culture System and CO_2_-Supplementation on Growth and Phosphate Uptake of SADW-Grown *Tolypothrix* sp.

**FIGURE 1:**
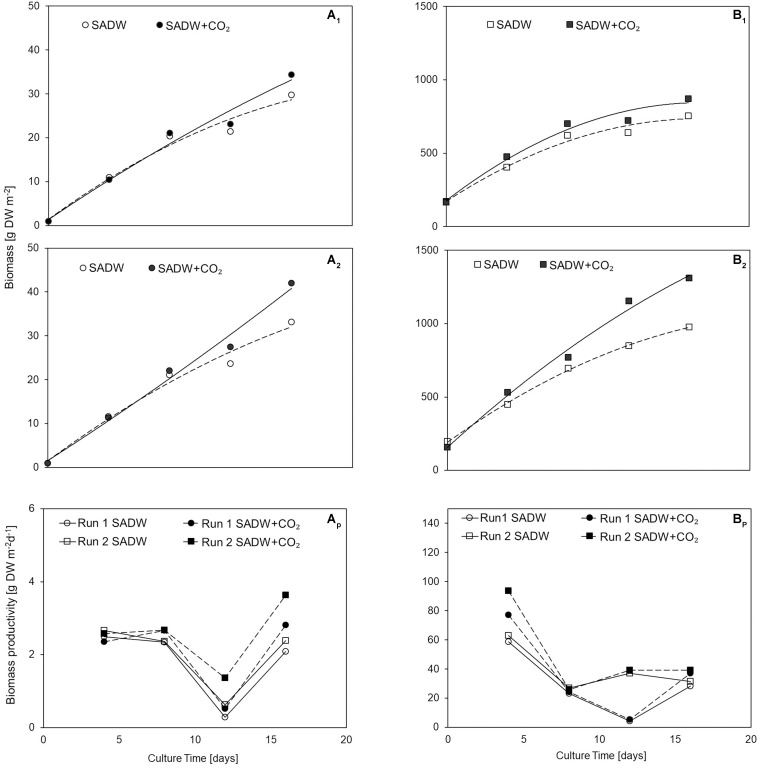
Effect of heavy metal and CO_2_ on *Tolypothrix* sp. growth in outdoor cultivation **(A_1_)** ATS run 1, **(A_2_)** ATS run 2, **(Ap)** ATS biomass productivity, **(B_1_)** V. Bag run 1, **(B_2_)** V. Bag run 2, **(Bp)** V. Bag biomass productivity.

**FIGURE 2:**
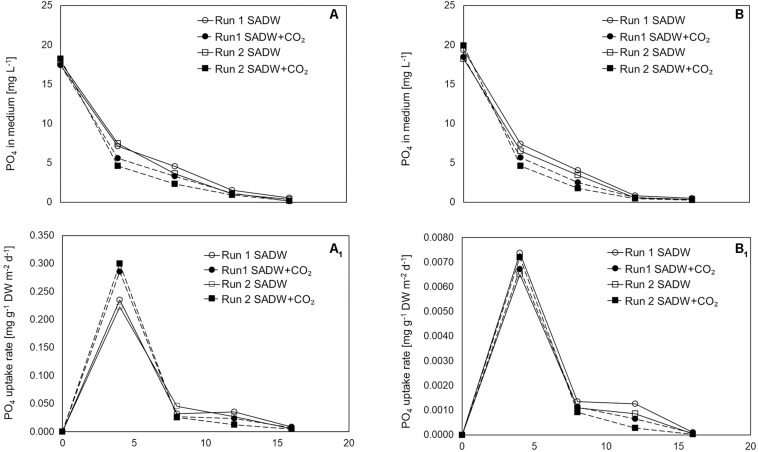
Effect of heavy metal and CO_2_ on culture medium phosphate levels and uptake rate of *Tolypothrix* sp. in outdoor cultivation. **(A,A_1_)** ATS and **(B,B_1_)** V. Bag.

CO_2_-supplementation enhanced growth of *Tolypothrix* sp. in both types of outdoor cultivation systems. A 1.15- and 1.26-times higher final biomass yield of 34 and 42 g DW m^–2^ was obtained for biofilm cultures (**Figure 1A_1,__2_**), while a 1.2- and 1.3-times higher yield in vertical suspension-based systems achieved a final biomass yield of 870 and 1310 g DW m^–2^ for runs 1 and 2, respectively (Figure 1B_1,__2_). Growth over the 16-day time course could be divided into two phases of specific growth rate (μ_1,2_) (Table 1). μ_1_ was not affected by CO_2_-supplementation for any of the cultivation systems, whereas μ_2_ was ∼21 and 30% higher for biofilm cultures and ∼11 and 32% higher for vertical bag suspension cultures for runs 1 and 2, respectively, when CO_2_ was supplied. The difference in μ_2_ between the two systems is not surprising, as CO_2_ requirements of the biofilms are supplemented by direct access to CO_2_ from the atmosphere. As such, growth in the bubble column suspension system appears to be light- and CO_2_-limited in dense cultures. CO_2_-supplementation had no effect on doubling rates (k) in either cultivation system, but doubling time was generally reduced by 15% for suspension-grown *Tolypothrix* sp. Despite positive outcomes of CO_2_-fertilization on biomass productivity (Figures 1A_p,_ B_p_) and system-dependent differences in growth performance, phosphate removal from the medium were slightly higher when supplemented with CO_2_, showing rapid uptake for the first 3 days of cultivation (Figures 2A,B). In contrast, phosphate removal rates (mg PO_4_^3–^ g^–1^ DW d^–1^) were higher for CO_2_-supplemented suspension cultures, while no trends were discernible for biofilms (Figures 2A_1_,B_1_). Growth of *Tolypothrix* sp. was phosphate-limited from day 8, and systems were devoid of phosphate from days 16 and 12 for CO_2_-supplemented cultures and non-CO_2_ controls of biofilm and suspension cultures, respectively. Biomass-standardized phosphate uptake rates were 0.2 to 0.3 for biofilms, but only 0.0065 to 0.0074 mg PO_4_^3–^ g^–1^ DW d^–1^ for suspension cultures and uptake rates were much lower for both cultivation systems from days 8 to 16, reflecting phosphate depletion from the SADW medium.

**TABLE 1:** Effect of CO_2_ and heavy metals on growth of *Tolypothrix* sp. in outdoor cultivation.

	Outdoor cultivation system							
	
	Algal turf-scrubber				Vertical bag			
		
	SADW		SADW + CO_2_		SADW		SADW + CO_2_	
				
	Run 1	Run 2	Run 1	Run 2	Run 1	Run 2	Run 1	Run 2
Specific growth rate (μ_1_) [d^–1^]	0.377	0.381	0.381	0.386	0.162	0.159	0.180	0.199
(μ_2_) [d^–1^]	0.048	0.057	0.061	0.081	0.024	0.045	0.027	0.066
Doubling rate (k) [d^–1^]	0.543	0.550	0.549	0.558	0.234	0.229	0.260	0.287
Doubling time (t_2_)	1.841	1.818	1.820	1.793	4.268	4.371	3.840	3.482

### Effect of Culture System and CO_2_-Supplementation on the Biochemical Profile of SADW-Grown *Tolypothrix* sp.

#### Carbohydrate, Protein, Lipid, Phycocyanin and Phycoerythrin Contents

**FIGURE 3:**
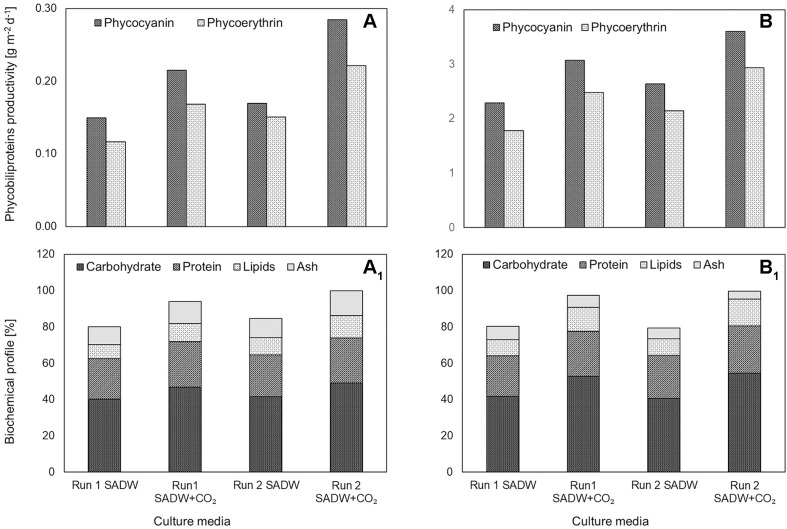
Effect of heavy metal and CO_2_ on pigments and biochemical profile of *Tolypothrix* sp. in outdoor cultivation. **(A,A_1_)** ATS and **(B,B_1_)** V. Bag.

CO_2_-supplementation increased carbohydrate and lipid contents of *Tolypothrix* sp. by 16 and 25% for biofilms and 26 and 38% for suspension cultures for runs 1 and 2, respectively (Figures 3A_1,_B_1_). In contrast, effects of CO_2_-supplementation on protein contents were marginal (Figures 3A_1,_B_1_). Maximal carbohydrate, protein and lipid contents were ∼49.2, 25.1, and 12.4% of cell dry weight (DW) for *Tolypothrix* sp. biofilms and ∼54.7, 26.0, and 14.8% of DW for suspension cultures when fertilized with CO_2_ (Figures 3A_1,_B_1_). Similarly, fertilization with 15% CO_2_ (v/v) increased phycobiliprotein (phycocyanin, phycoerythrin) contents (% w/w) by ∼40 and 27% for *Tolypothrix* sp. biofilms and suspension cultures, respectively. Maximal phycocyanin and phycoerythrin productivities were 0.3, 0.2, 3.6, and 2.9 g m^–2^ d^–1^ for biofilms and suspension cultures, respectively (Figures 3A,B).

#### Fatty Acid and Elemental Composition

As growth, lipid and phycobiliprotein contents were increased under CO_2_-fertilization, potential effects on fatty acid profiles and elemental composition (C, H, N, S, P, and K) and C/N ratios were investigated. Total fatty acid (TFA) contents and TFA productivities were ∼19 and 12% higher under CO_2_ supply for *Tolypothrix* sp. biofilms and suspension cultures, respectively. Maximal TFA yields were ∼75 mg g^–1^ DW for biofilms and ∼38 and 47 mg g^–1^ DW for runs 1 and 2, respectively (Table 2).

**TABLE 2:** Effect of CO_2_ and heavy metals on fatty acid profiles of *Tolypothrix* sp. in outdoor cultivation.

Fatty acids [mg g^–1^ DW]	Outdoor cultivation system							
	
	Algal turf-scrubber – biofilm				Vertical bag			
		
	SADW		SADW + CO_2_		SADW		SADW + CO_2_	
				
	Run 1	Run 2	Run 1	Run 2	Run 1	Run 2	Run 1	Run 2
14:1 (*cis-*9)	8.6	6.0	9.6	12.2	1.4	1.7	1.0	1.0
14:0	6.0	7.5	0.7	8.6	1.6	1.5	0.9	1.9
16:1	9.8	9.5	7.7	20.1	3.9	3.7	0.9	0.9
16:0	13.0	14.2	11.8	13.1	8.0	11.0	10.9	14.9
18:3 (*cis-*6, 9,12)	2.2	4.9	0.2	0.2	1.2	1.7	1.5	1.5
18:3 (*cis-*9,12,15)	10.1	7.1	18.5	13.0	1.4	4.7	6.1	8.1
18:2 (*cis*/*trans*-9,12)	1.0	1.6	8.9	0.4	2.6	3.3	4.4	4.5
18:1 (*cis*/*trans*-9)	3.0	2.7	7.6	3.4	3.0	5.0	4.9	6.9
18:0	0.2	0.2	0.5	0.2	1.2	1.3	1.2	1.8
SFA [mg g^–1^ FA]	20.7	22.9	14.9	23.8	13.5	17.5	15.1	20.0
MUFA [mg g^–1^ FA]	25.8	18.5	23.1	36.6	8.7	10.7	7.4	9.4
PUFA [mg g^–1^ FA]	13.4	14.6	33.7	13.5	8.9	12.8	14.8	17.9
Total Fatty acids [mg g^–1^ DW]	59.9	60.1	74.6	73.9	31.0	41.0	38.3	47.3
FA productivity [g g^–1^ DW m^–2^ d^–1^]	0.2	0.1	0.2	0.3	1.1	1.3	1.4	1.9

A positive effect of CO_2_-supplementation on saturated – (SFA), mono-unsaturated – (MUFA) and polyunsaturated fatty acid (PUFA) contents was noticeable for suspension-grown *Tolypothrix* sp., where the fatty acid profile was dominated by SFA, followed by PUFA and MUFA (Table 2). In contrast, responses to CO_2_-fertilization varied between both runs for *Tolypothrix* sp. biofilms, especially for MUFA and PUFA (Table 2), possibly due to variations in co-habiting bacterial communities, which are present and required for biofilm establishment and stabilization. The most abundant fatty acids were palmitic (hexadecanoic) acid (C16:0), followed by the ω-3-group of fatty acids α-linolenic acid [C18:3 (*cis* 9. 12. 15)], myristoleic acid (C14:1), the SFA myristic acid (C14:0), linoleic acid [C18:2 (cis/*trans* 9, 12)], the ω-9 oleic acid (C18:1) and the ω-6 γ-linolenic acid [C18:3 (*cis* 6, 9, 12)] (Table 2).

CO_2_-fertilization increased contents of C14:1, palmitoleic acid (C16:1), α-linolenic acid [C18:3 (*cis* 9. 12. 15)], C18:2 and C18:1 by 33, 31, 45, 72 and 48% in *Tolypothrix* sp. biofilms, respectively, while contents of C16:0 and C18:0 were unaffected. In contrast, CO_2_-supplementation increased C16:0, C18:3, C18:2, and C18:1 by 25, 57, 33 and 32% for *Tolypothrix* sp. suspension cultures, respectively (Table 2).

Culture system and CO_2_-supplementation did not result in large differences in C, H, N, S, P, and K contents. Carbon [∼45 and 47, and ∼47% (w/w)], K [∼0.79, 0.99, 0.68, and 0.79% (w/w)], and S [0.5, 0.7, and 0.7% (w/w)] were higher when supplemented with CO_2_ for *Tolypothrix* sp. biofilms and suspension cultures in runs 1 and 2, respectively (Table 3). A small positive effect of CO2-fertilization on nitrogen content and C/N ratios was also evident for *Tolypothrix* sp. biofilms, but not for suspension cultures (Table 3). In contrast, CO_2_ supply had a positive effect on P content of suspension cultures, but not biofilms (Table 3).

**TABLE 3:** Effect of CO_2_ and heavy metals on the elemental composition of *Tolypothrix* sp. in outdoor cultivation.

	Outdoor cultivation system							
	
	Algal turf-scrubber - biofilm				Vertical bag - suspension			
		
Elements [%]	SADW		SADW + CO_2_		SADW		SADW + CO_2_	
				
	Run 1	Run 2	Run 1	Run 2	Run 1	Run 2	Run 1	Run 2
Carbon (C)	41.21	44.38	45.12	47.0	43.17	44.53	47.43	47.47
Hydrogen (H)	6.42	7.18	7.07	7.03	6.99	7.21	7.10	7.13
Nitrogen (N)	6.63	6.88	7.18	7.01	7.18	7.33	7.05	7.06
Sulfur (S)	0.25	0.37	0.46	0.65	0.41	0.47	0.69	0.67
Phosphorous (P)	2.48	2.56	2.41	2.57	0.70	0.98	1.53	1.74
Potassium (K)	0.46	0.71	0.80	0.99	0.44	0.51	0.68	0.79
C/N ratio (C: N)	6.21	6.45	6.28	6.70	6.01	6.08	6.73	6.72

#### Metal Removal

A total of 16-day time course experiments investigated the effect of cultivation system and CO_2_ on metal removal from SADW, containing metals and concentrations typically found in ash dam water of coal-fired power plants, by *Tolypothrix* sp. (Table 4). Cultivation system and CO_2_-fertilization had no effect on maximal cumulative metal removal (Al, Sr, and Zn (≥90%), followed by Cu and Fe (∼70–80%), and As [∼65–75%)] for both biofilms and suspension cultures. In contrast, CO_2_-supplementation increased Mo removal by 37% in both cultivation systems, but an additional cultivation system effect was evident under CO_2_-fertilization, i.e., maximal Mo removal was ∼98% for biofilms but only ∼60% for suspension cultures. Conversely, a cultivation system effect was evident under CO_2_ supply for Se removal, with a 73% increase but a slight decrease for suspension-cultivated *Tolypothrix* sp. and biofilms, respectively. An even stronger cultivation effect was observed for Ni, where CO_2_-supplementation negatively affected removal in biofilm cultures, but not suspension cultures of *Tolypothrix* sp.

**TABLE 4:** Effect of CO_2_ on cumulative metal removal from SADW medium by *Tolypothrix* sp. over a 16-day time course.

Metals	Initial concentration [μg L^–1^]	Metal removal in outdoor cultivation system [%]							
		
		Algal turf-scrubber - biofilm				Vertical bag - suspension			
			
		SADW		SADW + CO_2_		SADW		SADW + CO_2_	
					
		Run 1	Run 2	Run 1	Run 2	Run 1	Run 2	Run 1	Run 2
Al	200.00	99.8	99.8	99.8	99.8	99.8	99.8	99.8	99.8
As	31.60	70.0	68.4	69.0	69.0	67.4	63.5	75.7	75.7
Cu	38.20	83.2	82.3	79.5	74.3	76.2	72.5	77.2	77.2
Fe	1110.00	81.0	79.5	75.7	65.5	72.4	70.8	79.7	76.9
Mo	1040.00	63.6	58.9	98.9	98.9	38.9	27.5	60.3	60.3
Ni	22.90	80.1	79.1	57.5	57.5	69.6	63.7	68.3	54.6
Se	174.00	82.5	81.7	77.8	77.7	23.2	12.3	84.2	84.2
Sr	830.97	99.2	99.1	98.6	98.4	98.8	98.8	99.3	99.0
Zn	90.70	94.0	93.5	90.8	90.6	100.0	87.00	100.0	98.0

### Economic Viability Assessment of Bioproduct Commercialization Derived From SADW-Produced *Tolypothrix* sp. Biomass – Effect of Co-location With Coal-Fired Power Plants

#### Direct and Indirect Capital and Operational Costs

Based on the biomass productivities and biochemical profiles achieved with CO_2_ supplementation of SADW-grown *Tolypothrix* sp. in vertical bubble column suspension cultures, NPV analyses assessed the economic viability of four bioproduct scenarios under co-location with coal-fired power stations and traditional cultivation (no co-location) in raceway ponds, commonly used for production at commercial scale. The four bioproduct scenarios modeled were: (1) food-grade phycocyanin as the sole product, (2) biofertilizer as the sole product, (3) 50% of biomass used each for food-grade phycocyanin and biofertilizer production, and (4) biorefining of phycocyanin (100%) and use of the extracted biomass as a biofertilizer (Table 5). All capital costs and operating costs for *Tolypothrix* sp. cultivation (Table 5) were derived from published data for production of microalgal biomass (Davis et al., 2011; Griffin and Batten, 2013; Heimann et al., 2015; Schenk, 2016; Doshi, 2017; Fornarelli et al., 2017). Direct and indirect capital costs (engineering fees set at 15% of capital and contingency set at 5% of capital) for building a 10 ha production facility and some operational expenses were considered to be unavoidable. The co-location scenario considered savings on capital costs (land acquisition) and operational costs for maintenance and insurance, water (ash dam wastewater) and CO_2_ supply (flue gas) (Table 5), but did not apply potential income generated through CO_2_ emission reduction and wastewater treatment, as these were deemed covered by the savings made. Irrespective of location scenario, the lack of nitrogen-fertilization and the benefits of the self-settling properties of *Tolypothrix* sp. on energy saving for harvesting were considered by applying no costs for nitrogen fertilization and a 90% saving on dewatering costs (Table 5). Accordingly, total capital costs for constructing the 10 ha facility not co-located with a coal-fired power plant were estimated at US$ 853,442 for pond construction, CO_2_, nutrient supply, water-recirculation, and harvest/dewatering systems and land acquisition. The only direct capital cost avoided by co-location was for land acquisition (US$ 147,526), reducing the direct capital cost to US$ 705,916 (Table 5). Standardized indirect capital costs for engineering fees (including land acquisition), set at 15% and applying a 5% contingency were estimated to be US$ 170,688 for a not co-located facility, of which US$ 29,505 were avoided through free land provided in the co-location scenario, reducing indirect capital costs to US$ 141,183 (Table 5). A working capital of 5% of the total costs did not consider any of the benefits derived through co-location. Total annual operating costs for a not co-located facility was estimated to be US$ 2,440,633 for the 10 ha facility and included cost for salaries, maintenance and insurance, phosphate fertilizer and water requirements, CO_2_, energy for cultivation, dewatering and drying, and pigment extraction and purification (Table 5). Co-location resulted in significant operational savings of ∼70%, particularly through avoiding water and CO_2_ supply costs and a 90% saving on maintenance and insurance, and energy expenditure; the latter considered that energy would be purchased from the power station at 10% of the sales price to ordinary customers, and maintenance/insurance costs would also be 10% of ordinary costs. Therefore, annual operational costs were estimated to be US$ 758,241 when co-located (Table 5).

**TABLE 5:** Microalgal culturing facility capital and operating costs, product income potential, net profit value and sensitivity analyses.

Size of *Tolypothrix* facility [ha]	10		
**Microalgal culturing facility costs**			
**Direct unavoidable capital costs**	**Units**	**Cost (USD) ha**^–^**^1^**	**Cost (USD) 10 ha**^–^**^1^**
Open raceway pond construction	$ ha^–1^	35,436	354,368
CO_2_ feed system	$ ha^–1^	6,717	67,170
Water and nutrient system	$ ha^–1^	15,832	158,321
Harvesting and dewatering system	$ ha^–1^	12,606	126,057
**Total unavoidable direct capital cost**	**$**		**705,916**

**Direct capital costs avoided by co-location**			147,526
Land acquisition Co-located (assumed land owned by partner industry)	$ ha^–1^	14,753	
**Total avoidable capital cost**	**$**		**147,526**

***Grand total of all capital cost***	***$***		***853,442***

**Standardized indirect capital costs**			
Engineering Fees – unavoidable	15% capital		105,887
Land acquisition fees – avoidable	15% capital		22,129
**Engineering fees – total**	**15% capital**		**128,016**
Contingency – unavoidable	5% capital		35,296
Contingency – avoidable	5% capital		7,377
Contingency – total	5% capital		**42,673**
**Total indirect costs - unavoidable**	**$**		**141,183**
**Total indirect costs - avoidable**	**$**		**29,505**

***Total indirect costs***	***$***		***170,688***
**Working capital - all unavoidable**	**5%capital**		**51,206**

**Operational costs**			
**Operational costs not co-located**	**Units**		
Labor-plant manager/supervisor	1 person ($)		81,072
Labor-Engineer	1 person ($)		60,333
Labor-Lab analyst	2 persons ($)		90,499
Labor-Administration	1 person ($)		43,364
Labor-Technician/pond operator	2 persons ($)		71,645
Maintenance and insurance	10%^B^		84,710
Phosphate input	$ t^–1^		35,757
Water requirement-avoidable if co-located with waste water industry	$	1,217,646	1,217,646
CO_2_ purchase - unpaid able if co-located with coal fired power stations	$	397,127	397,127
Energy demand cultivation, dewatering and drying	$ y^–1^	10,980	10,980
Costs for pigment extraction and purification^*A*^	$ t^–1^ biomass		347,500
**Total annual operating costs when not co-located**	$		**2,440,633**

**Operational costs when co-located**			
Labor-plant manager/supervisor	1 person ($)		81,072
Labor-Engineer	1 person ($)		60,333
Labor-Lab analyst	2 persons ($)		90,499
Labor-Administration	1 person ($)		43,364
Labor-Technician/pond operator	2 persons ($)		71,645
Maintenance and insurance	10%^B^		17,703
Phosphate input	$ t^–1^		35,757
Ash dam water	$		0
Flue gas	$		0
Energy demand for flue gas supply (at 10% of supply charge to customer)	$ y^–1^	9,270	9,270
Energy demand cultivation, dewatering and drying (at 10% of supply charge to customer)	$ y^–1^	1,098	1,098
Costs for pigment extraction and purification^*A*^	$ t^–1^ biomass		347,500
**Total annual operating costs when co-located**	**$**		**758,241**

**Bioproduct income**			
**Biomass derived potential income**			
Food-grade phycocyanin 10.8 t ha^–1^ y^–1^ (US$ 500 kg^–1^) at an extraction/purification efficiency of 67%^A^	$ ha^–1^ y^–1^	3,448,155	34,481,550
Biofertilizer/Biochar (US$ 500 t^–1^ DW) (117.5 t biomass dry weight)	$ ha^–1^ y^–1^	58,750	587,500
Biofertilizer from 50% biomass (US$ 500 t^–1^ DW) (58.57 t biomass dry weight)	$ ha^–1^ y^–1^	29,375	293,750
**Weighted average costs of capital (WACC)**	**%**		**10**

**Net profit value analyses**			
***Profit value (PV) scenarios over a 20-year period***		**Co-located**	**Not co-located**
1) Food-grade phycocyanin (sole product)	$	538,038,679	491,380,960
2) 100% of biomass converted to biofertilizer (sole product)	$	−12,294,786	−58,952,5056
3) 50% of biomass converted to biofertilizer +50% food-grade phycocyanin	$	273,711,820	250,382,961
4) 100% food-grade phycocyanin and biofertilizer yields	$	547,423,641	500,765,922
***Sales tax and distribution costs for PV scenarios 1–4***			
1) Assumed tax and distribution costs including transport as % sales value (50%)	$	269,019,340	245,690,480
2) Assumed tax and distribution costs including transport as % sales value (50%)	$	0	0
3) Assumed tax and distribution costs including transport as % sales value (50%)	$	136,855,910	125,191,480
4) Assumed tax and distribution costs including transport as % sales value (50%)	$	273,711,820	250,382,961
**Net profit value for scenarios 1–4**			
1) Food-grade phycocyanin (sole product)	$	269,019,340	245,690,480
2) 100% of biomass converted to biofertilizer (sole product)	$	−12,294,78	−58,952,50
3) 50% of biomass converted to biofertilizer +50% food-grade phycocyanin	$	136,855,910	125,191,480
4) 100% food-grade phycocyanin and biofertilizer yields	$	273,711,820	250,382,961

**Sensitivity Analyses for NPV scenarios 1–4**			
***Biofertilizer price at 25% (125 USD t***^–^***^1^)***			
1) Food-grade phycocyanin (sole product)	$	269,019,340	245,690,480
2) 100% of biomass converted to biofertilizer (sole product)	$	0	0
3) 50% of biomass converted to biofertilizer +50% food-grade phycocyanin	$	135,244,045	123,431,800
4) 100% food-grade phycocyanin and biofertilizer yields	$	270,488,090	246,863,600
***Food-grade phycocyanin price at 25% (125 USD kg***^–^***^1^)***			
1) Food-grade phycocyanin (sole product)	$	59,067,104	35,738,244
2) 100% of biomass converted to biofertilizer (sole product)	$	0	0
3) 50% of biomass converted to biofertilizer +50% food-grade phycocyanin	$	32,471,052	20,215,362
4) 100% food-grade phycocyanin and biofertilizer yields	$	64,942,104	40,430,725

**Net Profit Value and Sensitivity Analysis For Raceway Production Scenario for Biomass Production at 6 t DW ha**^–^**^1^ y**^–^**^1^**			

**Net profit value analyses**			
***Profit Value (PV) scenarios over a 20-year period***			
1. Food-grade phycocyanin (sole product)	$	264,058,457	217,400,737
2. 100% of biomass converted to biofertilizer (sole product)	$	−16,962,887	−63,620,607
3. 50% of biomass converted to biofertilizer +50% food-grade phycocyanin	$	136,721,709	113,392,849
4. 100% food-grade phycocyanin and biofertilizer yields	$	273,443,418	226,785,699
***Sales tax and distribution costs for PV scenarios 1–4***			
1. Assumed tax and distribution costs including transport as % sales value (50%)	$	132,029,228	108,700,369
2. Assumed tax and distribution costs including transport as % sales value (50%)	$	0	0
3. Assumed tax and distribution costs including transport as % sales value (50%)	$	68,360,855	56,696,425
4. Assumed tax and distribution costs including transport as % sales value (50%)	$	136,721,709	113,392,849
**Net profit value for scenarios 1–4**			
1. Food-grade phycocyanin (sole product)	$	132,029,228	108,700,369
2. 100% of biomass converted to biofertilizer (sole product)	$	−16,962,887	−63,620,607
3. 50% of biomass converted to biofertilizer +50% food-grade phycocyanin	$	68,360,855	56,696,425
4. 100% food-grade phycocyanin and biofertilizer yields	$	136,721,709	113,392,849

**Sensitivity Analyses for NPV scenarios 1–4**			
***Biofertilizer price at 25% (125 USD t***^–^***^1^)***			
1. Food-grade phycocyanin (sole product)	$	132,029,228	108,700,369
2. 100% of biomass converted to biofertilizer (sole product)	$	−20,616,184	−67,273,904
3. 50% of biomass converted to biofertilizer + 50% food-grade phycocyanin	$	68,360,855	56,696,425
4. 100% food-grade phycocyanin and biofertilizer yields	$	136,721,709	113,392,849
***Food-grade phycocyanin price at $125 kg^–1^***			
1. Food-rade phycocyanin (sole product)	$	24,819,576	1,490,716
2. 100% f biomass converted to biofertilizer (sole product)	$	−16,962,887	−63,620,607
3. 50% of biomass converted to biofertilizer +50% food-grade phycocyanin	$	14,756,028	3,091,598
4. 100% food-grade phycocyanin and biofertilizer yields	$	29,512,057	6,183,197

*^*A*^(Chaiklahan et al., 2018); ^*B*^Total direct and indirect capital.*

#### Bioproduct-Generated Income, Net Present Value and Sensitivity Analyses

Bioproduct yields were estimated from biomass productivity and bioproduct productivities obtained in this study and for average productivities achieved for *Nannochloropsis* occulata in large-scale outdoor raceway cultivation. Based on this, it is estimated that a 10 ha facility could produce 117.5 and 60 t *Tolypothrix* sp. biomass ha^–1^ y^–1^ for a 300-day production period, respectively. The average phycocyanin content of *Tolypothrix* sp. used in this study is 8.8% (w/w), although as shown here, higher yields are possible. In order to put the NPV on a realistic footing, the average yields were used to calculate the yields of food-grade phycocyanin (A620 nm/A280 nm: 0.7), which can be extracted and purified with an efficiency of 67% (Chaiklahan et al., 2018). This equates to a production of 10.3 t unpurified phycocyanin ha^–1^ y^–1^, which yields 6.9 t food-grade phycocyanin ha^–1^ y^–1^, valued at US$ 3,448,155 ha^–1^ y^–1^ based on a sales price at US$ 500 kg^–1^ (Table 5). The final purified product will require metal analysis for quality assurance, which has not been considered as a cost, as costs for these analyses are expected to be absorbed by the coal-fired power plant the production would be collocated with. Furthermore, production of the food-grade phycocyanin produced when not collocated would not be subject to such analyses, i.e., in this scenario no metal analyses costs would be incurred. Biofertilizer/biochar can fetch a sales price of US$ 500 t^–1^, which, based on biomass productivities achieved here, equates to US$ 58,750 ha^–1^ y^–1^ (Table 5). Based on this, the predicted values for the modeled bioproduct scenarios are highest for co-located production for phycocyanin extraction in a biorefinery approach and conversion of the extracted biomass to biofertilizer (scenario 4, US$ 547,423,641), closely followed by producing food-grade phycocyanin as the sole product (scenario 1, US$ 538,038,679) (Table 5). Net present values were 50% lower based on assumed tax and distribution costs (Table 5).

In order to decide whether a project remains commercially viable, sensitivity analyses are essential. Accordingly, the weighted average costs of capital (WACC) is 10% and the sensitivity analyses modeled two scenarios: (1) reduction of the biofertilizer price to 25% of the current value (US$ 125 t^–1^) and (2) a food-grade phycocyanin price to 25% of the current value due to market saturation (US$ 125 kg^–1^). This showed that producing biofertilizer as the sole product (scenario 2) is not commercially viable even when the production facility is co-located. It is assumed that income for environmental services provided at the coal-fired power plant would not provide a strong business incentive. In contrast, producing phycocyanin as the sole product (scenario 1) remains to be of commercial interest (Table 5).

## Discussion

### Effect of CO_2_-Supplementation on Biomass Production

Successful cultivation of microalgae and cyanobacteria in wastewaters arising from energy -, mining - and mineral-processing industries would provide for a sustainable platform for production of algal biomass and bioproducts, whilst simultaneously providing for efficient bioremediation of potentially harmful nutrients and metals (Roberts et al., 2013). Significant growth challenges, hampering overall productivities and economics when using wastewater for production, must be addressed first for the realization of the true commercial potential.

This study demonstrated that CO_2_ supplementation significantly improved final phycobiliprotein and biomass yields, as well as biomass productivities of the diazotrophic cyanobacterium *Tolypothrix* sp. grown in SADW under outdoor conditions as biofilms of suspension cultures. Similar increases of up to 60% have been reported for biomass productivities using the non-diazotrophic cyanobacterium *Arthrospira platensis* for cultures supplemented with 1% CO_2_ (v/v) (Ravelonandro et al., 2011). In contrast, growth of *Tolypothrix* sp. was reduced by 60% under outdoor (this study) compared to indoor suspension cultivation under the same CO_2_-supplemented – and wastewater conditions (Velu et al., 2019). Growth of outdoor cultures is often generally lower due to difficulties in controlling cultivation and environmental conditions, such as hydrodynamics, temperature, UV irradiation and irradiance within optimal ranges (Chen et al., 2011). Light intensity is one of the major factors affecting cyanobacterial and microalgal growth. The optimal light intensity for growth of *Tolypothrix* sp. is ∼500 μmol photons m^–2^ s^–1^, however, observed light intensities at noon varied from 500 to 900 μmol photons m^–2^ s^–1^ (Supplementary Table S3). This could suggest that periodic photoinhibition might have occurred, especially in younger cultures with low cell concentrations. It must be emphasized that the reported CO_2_-supplemented growth performance was comparable to other microalgae and cyanobacteria grown in various cultivation systems but was achieved without nitrogen-fertilization (Table 6). In addition, biomass growth was phosphate-limited after ∼3 to 8 days of cultivation. It is therefore conceivable that phosphate fertilization at appropriate intervals should improve biomass yields and productivities further. High temperature and irradiance experienced in the ATS were the most likely factors impeding growth performance of biofilms of *Tolypothrix* sp. (Del Campo et al., 2007; Chen et al., 2011), a conclusion supported by higher growth performance of indoor-cultivated microalgal biofilms (Table 6).

**TABLE 6:** Effect of CO_2_ on the performance of cyanobacterial and microalgal cultivation systems.

Culturing mode	System	Species	Culture medium	CO_2_ [%]	Location	Cultivation system working volume/area	Biomass productivity [g m^–2^ d^–1^]	References
Suspension	Vertical flat-plate photobioreactor (PBR)	*Synechocystis aquatilis* SI-2	Modified SOT inorganic medium	10 40	Outdoor^1^	24 L 9 L	31–45 18.3	Zhang et al., 1999, 2001
	Bubble column PBR	*Anabaena* sp. ATCC 33047	Detailed in^a^	NG	Indoor^2^	9 L	81	López et al., 2009
	Acrylic cylindrical tank	*Phormidium valderianum*	TDS effluent^b^	NG	Outdoor^3^	550 L	0.03	Dineshbabu et al., 2017
	PBR	*Anabaena sp.* CH1	Arnen medium	15	Indoor^4^	5 L	∼31	Chiang et al., 2011
	PBR	*Chlorella* sp. MM-2	Modified f/2	5	Indoor^4^	0.8 L	∼51	Kao et al., 2012
	PBR	*Chlorella* sp. MM-2	Swine wastewater	20	Outdoor^4^	50 L	∼36	
	Column PBR	*Spirulina* sp. (*Arthrospira*)	Zarrouk medium	6 and 12	Indoor^5^	1.8 L	27 and 22	De Morais and Costa, 2007
		*Scenedesmus obliquus*	MC medium			1.8 L	12 and 17	
	Aerated vertical bag	*Tolypothrix* sp.	SADW^c^	15	Outdoor^6^	500 L	25–95	This study
Biofilm	Single layer PBR	*Botryococcus braunii*	Autotrophic nutrient medium	1	Indoor^7^	0.08 m^–2^	6.5	Cheng et al., 2013
	Multi-layer PBR					0.08 m^–2^	49.1	
	Twin-Layer PBR	*Halochlorella rubescens*	LSBM^d^	3 and 5	Indoor^8^	3 m^–2^	∼30 and 24	Schultze et al., 2015
	PBR	*Pseudochlorococcum* sp.	BG11	10	Indoor^7^	0.00025 m^–2^	∼5.5	Ji et al., 2014
	Algal turf-scrubber	*Tolypothrix* sp.	SADW	15	Outdoor^6^	2.2 m^–2^	1.36–3.63	This study

*^1^Japan; ^2^Spain; ^3^South India;^4^Taiwan;^5^ Brazil; ^6^Queensland, Australia; ^7^China; ^8^Germany. ^*a*^Culture medium detailed in Moreno et al. (1998); ^*b*^Seawater and ossein effluent from gelatin manufacturing industry;. ^*c*^Simulated ash-dam wastewater; ^*d*^ Large Scale Brackish Medium prepared based on Bold’s Basal medium NG- CO_2_ percentage not given but CO_2_ was used.*

The observed higher growth performance of *Tolypothrix* sp. when supplemented with CO_2_ could be attributable to positive effects on nitrogen – and photosynthesis-linked carbon fixation. For example, supplementation of cultures of the marine diazotrophic cyanobacterium *Trichodesmium* sp. with large amounts of CO_2_ resulted in a 20% increase in nitrogen fixation rates (Levitan et al., 2007). In addition, CO_2_ supplementation could have allowed for the reallocation of energy required for inorganic carbon (C_*i*_) uptake and scavenging of O_2_. For instance, CO_2_-supplementation reduced the energy requirements for C_*i*_ uptake in *Trichodesmium* sp. by suppressing the energy-intensive carbon concentrating mechanism (CCM), which are employed under carbon-limiting conditions, freeing up this energy for the fixation of atmospheric nitrogen, another metabolic pathway with large energy requirements (Levitan et al., 2007). In contrast, despite a large effect of CO_2_-supplementation on biomass productivity, no effect was observed for phosphate requirements in biofilm and suspension-grown *Tolypothrix* sp., which was also observed for indoor-cultivated suspension cultures (Velu et al., 2019). This could represent a direct result of phosphate limitation after large uptake over the first 3 days of the growth period for replenishing internal phosphate stores, providing sufficient energy for CO_2_ fixation. In the context of deploying industrial cultivation of *Tolypothrix* sp. for the bioremediation of macronutrient-poor ash dam wastewaters at coal-fired power plants, the ability of sustained growth without requirements for nitrogen-fertilization offers a distinctive economical advantage. For example, nitrate provision for large-scale production of the non-diazotrophic *Arthrospira platensis* (synonym *Spirulina platensis*) was estimated to account for 50% of the overall production costs (Vonshak and Richmond, 1988). Energy savings provided by the self-settling ability of *Tolypothrix* sp. is another significant advantage for wastewater-utilizing large-scale cultivation, as costly dewatering infrastructure and energy requirements, that apply to the commonly used microalgal genera *Chlorella* spp. and *Scenedesmus* spp. (Silva and Silva, 2007), is abolished. For example, biofilms and self-flocculated biomass of *Tolypothrix* sp. were 80- and 53-fold more concentrated than the original suspension culture (Velu et al., 2015). Taken together, these properties reduce the need for finite chemical fertilizers and improve the overall economics of cultivation in macronutrient-limited ash dam wastewaters.

### Effect of CO_2_-Supplementation on Biochemical Profiles, Metal Removal Capacity and Bioproduct Potential

CO_2_-fertilization of outdoor-grown biofilms and suspension cultures of *Tolypothrix* sp. resulted in increased total carbohydrate and lipid contents, as has also been reported for indoor cultivated suspension cultures (Velu et al., 2019). Similarly, an increase in CO_2_ supply from 5 to 25% (v/v) increased total carbohydrate and lipid contents of *Scenedesmus bajacalifornicus* by up to 20 and 10%, respectively (Patil and Kaliwal, 2017). Elevated CO_2_ supplies typically result in increased carbohydrate contents in microalgae, and are likely a result of enhanced photosynthetic efficiencies (Giordano, 2001) or CO_2_-induced low pH stress (Dragone et al., 2011). In contrast, 15% CO_2_ (v/v) supplementation led to an increase in pH from 6.0 to 9.0 and 7.0 to 9.0 for CO_2_-supplemented and non-CO_2_ controls under outdoor cultivation of *Tolypothrix* sp. biofilms and suspension cultures (Table 6), suggesting strongly that cultures were still carbon-limited (Coleman and Colman, 1981). Accounting for the fact that nitrogen-requirements for growth had to be met solely through nitrogen fixation, it is not surprising that CO_2_-supplementation had no effect on protein content for biomass cultivated in either system, which differs from other reported outcomes, where CO_2_-supplementation has been shown to correlate with improved nitrate uptake and thus higher protein production (Xia and Gao, 2005). As demonstrated by significant CO_2_-induced increases in the nitrogen-containing pigments phycocyanin and phycoerythrin, nitrogen supply through nitrogen fixation must have been sufficient under outdoor cultivation of *Tolypothrix* sp. The increase in the content of these pigments could have been also responsible for improved growth performance under CO_2_-supplementation, as they are accessory pigments for the capture of light in the light harvesting complexes of the photosystems and protect the photosynthetic apparatus from excess light and reactive oxygen damage (Chakdar and Pabbi, 2016).

Similar to the increase in phycobiliprotein contents, i.e., phycocyanin and phycoerythrin, CO_2_-supplementation resulted in 19 and 12% higher TFA contents in *Tolypothrix* sp. biofilms and suspension-grown biomass, which is similar to results obtained in indoor cultivation (Velu et al., 2019) and with eukaryotic microalgae (Tsuzuki et al., 1990), but no significant effect on MUFA or PUFA content was evident. α- (C18:3 ω-3) and γ-Linolenic acid (C18:3 ω-6) are important dietary supplements with critical health benefits, and the latter is also an ingredient in cosmetics (Ryckebosch et al., 2012). Similar to indoor suspension cultivation (Velu et al., 2019), *Tolypothrix* sp. produced 25% C18:3 ω-6 or 18.5 mg g^–1^ TFA, which is higher than reported for *Arthrospira* (*Spirulina*) sp. (11–16%) (De Oliveira et al., 1999). In contrast to phycocyanin, yields of (C18:3 ω-6), however, remained insufficient for consideration as a main target product in a biorefinery approach, due to low TFA contents characteristic for cyanobacteria.

Irrespective of cultivation system and CO_2_-fertilization, metal bioremediation of *Tolypothrix* sp. from macronutrient-poor ash dam wastewater, containing concentrations of Al, As, Cd, Ni, and Zn that exceed the ANZECC guidelines (Roberts et al., 2013), showed that levels were lowered to acceptance thresholds at the end of the cultivation period. This demonstrated that *Tolypothrix* sp. is a suitable organism for the bioremediation of metals from complex mixtures, under macronutrient-limiting conditions. The produced *Tolypothrix* sp. biomass was rich in carbon (45%) and nitrogen (7%), resulting in a C/N ratio of 6.58, similar to results obtained in indoor cultivation (Velu et al., 2019). In addition, the elemental composition and concentrations were comparable to those found in other cyanobacteria, previously reported as suitable for biofertilizer applications (Osman et al., 2010). Importantly, biomass of *Tolypothrix* sp. remained suitable at application rates required for the fertilization of wheat, supplying in addition to nitrogen and carbon also essential trace elements, such as Cu, Fe, Mo, and Zn (Velu et al., 2019). Diazotrophic cyanobacteria, such as *Tolypothrix* sp., are natural and renewable sources of biological nitrogen, contributing up to 30 kg N ha^–1^ and providing large quantities of organic matter and important plant hormones (i.e., gibberellin, auxin and cytokines) to soils, thereby improving soil fertility (Issa et al., 2014), supporting plant development and protecting against pathogens (Singh et al., 2016). Diazotrophic cyanobacteria, such as *Tolypothrix* sp., are commonly employed as biofertilizers in rice fields (Karthikeyan et al., 2007) and applications of *Tolypothrix* sp. specifically resulted in a 25% increase in crop yields in poorly drained rice fields (Watanabe et al., 1951). Long-term applications improved nitrogenous fertility of soils, attributable to the increase in soil carbon and nitrogen through accumulation of decomposing and live biomass, respectively (Watanabe, 1962). In summary, the above application potential, together with CO_2_-enhanced growth responses and phycocyanin yields, makes *Tolypothrix* sp. biomass production for use as a biofertilizer a real potential in regions, where agricultural production is located near freshwater-using coal-fired power plants, as is the case for the Tarong power station in Queensland, Australia.

To test and substantiate the commercial viability of *Tolypothrix* sp.-derived food-grade phycocyanin and biofertilizer, net present value and sensitivity analyses evaluated four bioproduct scenarios for the production of *Tolypothrix* sp. biomass under coal-fired power plant co-location and non-colocation of the production facility. Production in traditional raceway ponds was chosen, as no published data on the construction costs of large-scale production facilities using bubble columns could be found and a comparison of average microalgal and cyanobacterial maximal biomass productivities was not strongly influenced by cultivation system when operated under outdoor conditions (Heimann et al., 2015). In addition, biofilm cultivation was not considered in the modeled scenarios for several reasons. (1) Systems used in the study have a very large areal production footprint. (2) The areal productivity is low. (3) The systems are more prone to contamination by other microalgae when used for extended periods under outdoor conditions (Velu et al., 2015). (4) Establishment costs for large-scale production cannot be applied with any certainty, as biofilm cultivation systems vary significantly in design (Heimann, 2016). (5) As the biofilms were not harvested at regular intervals, it is impossible to determine the true yield potential of the systems at this stage, as regrowth of the remaining turf may have vastly different biomass production characteristics compared to freshly seeded turfs.

The modeled NPV and sensitivity analyses showed that the production of food-grade phycocyanin is advantageous for commercial viability, whether or not the facility would be co-located, whereas biofertilizer production as a sole product was not commercially viable in any of the modeled scenarios. Outcomes for biofertilizer income were similar to the commercial production of *Azospirillum*, a nitrogen-fixing bacterium, simulated for liquid biofertilizer production in Cuba, but the production scale for the plant was 4-fold larger in terms of product volume (Pérez Sánchez et al., 2018) than for the *Tolypothrix* sp. plant in the presented study. In that analysis, salary costs accounted for >50% of the production cost, as the process is labor-intensive, requiring 29 employees to man 24 h shifts (Pérez Sánchez et al., 2018), while production of *Tolypothrix* sp. biomass represented only 10% of the overall production costs. An NPV analysis for the commercial production of dried microalgal biomass (US$625 t^–1^) using dairy effluent as a nutrient and water supply also concluded that the process is commercially feasible for a plant size treating 1 million liter of dairy effluent over a 20-year period (Kumar et al., 2020). The sensitivity analyses using one quarter of today’s food-grade phycocyanin sales price demonstrated that facilities producing phycocyanin as a sole product or phycocyanin and biofertilizer remain commercially viable whether co-located or not. Instead of using product sales prices, reduction of biomass yields are an alternative parameter in sensitivity analyses. Reduction of biomass yields to one quarter of the original tonnage therefore had a comparable effect on NPV outcomes (Kumar et al., 2020). An obvious worst-case scenario for commercial production would be reduced yearly biomass yields (reduced to 33%) and reduced product sales prices (at 25% each of today’s sales prices). Applying this situation to the *Tolypothrix* sp. production scenario proposed here over the entire 20-year period determined that production of food-grade phycocyanin as a sole product, 50% of biomass extraction for each food-grade phycocyanin and biofertilizer, and 100% food-grade phycocyanin with the residual biomass converted to biofertilizer (100%) remain commercially feasible irrespective of co-location or not for the bubble column production scenario, but only when co-located for the raceway production simulation. Therefore, with regards to a decision whether co-location offers significant benefits, the profit difference predicted here would be as large as ∼US$ 23 million over 20 years for production of 100% food-grade phycocyanin at a quarter of today’s sale price with 100% co-production of biofertilizer. This provides a significant incentive for co-locating production facilities with CO_2_-polluting and metal-rich wastewater generating industries, for the simultaneous application of the environmental services of diazotrophic cyanobacteria and bioproduct development.

## Conclusion

This study demonstrated significantly enhanced biomass and phycocyanin yields and productivities in response to CO_2_-fertilization and excellent metal removal capacity of *Tolypothrix* sp. cultivated under outdoor conditions in meso-scale systems without supply of nitrogen fertilization. This makes *Tolypothrix* sp. an outstanding candidate for bioremediation of CO_2_ and metals at freshwater-utilizing coal-fired power plants. Obtained growth performance suggests that 10.91 and 117.5 t dry biomass ha^–1^ can be produced in a year set at 300 days of cultivation in biofilms and vertical bag suspension cultures, respectively. The NPV and sensitivity analyses performed with production data obtained in this study and modeled for a simulated raceway production scenario, taking the organism’s self-settling ability into account, demonstrated that co-location with coal-fired power plants was not essential for commercial viability, but significantly increased achievable net present values for all modeled product scenarios, making it an attractive proposition, if freshwater-utilizing plants are in close proximity to agricultural land. The most profitable scenario was production of food-grade phycocyanin (100%) coupled with co-production of biofertilizer (100%), followed by food-grade phycocyanin as the sole product, and 50% of each phycocyanin and biofertilizer production. In contrast, production of biofertilizer as a sole product was not commercially viable under any of the modeled scenarios. Based on the above, cultivation of *Tolypothrix* sp. in vertical suspension cultures with CO_2_ supply, but without nitrogen-fertilization is recommended for the production of food-grade phycocyanin either as a sole product or with co-production of biofertilizer.

## Data Availability Statement

The raw data supporting the conclusions of this article will be made available by the authors, without undue reservation, to any qualified researcher.

## Author Contributions

KH, SC, and CV were responsible for the experimental design of this study and analyzed all the data. CV carried out the experimental work of the outdoor growth experiments, protein – and phycobiliprotein contents. DB and CV conducted the analysis and quantification of biomass lipid content and fatty acid profiles. KH and CV performed the NPV and sensitivity analyses. KH, CV, SC, and DB jointly assembled and critically interpreted these data for publication. All authors contributed to writing the manuscript, provide approval for publication, and agreed to be accountable for all aspects of the work.

## Conflict of Interest

The authors declare that the research was conducted in the absence of any commercial or financial relationships that could be construed as a potential conflict of interest.
