# Supplementary material for: Bioproduct Potential of Outdoor Cultures of Tolypothrix sp.: Effect of Carbon Dioxide and Metal-Rich Wastewater
Source: Front Bioeng Biotechnol. 2020 Feb 11;8:51. doi: 10.3389/fbioe.2020.00051 (PMC7026013; doi:10.3389/fbioe.2020.00051)
Supplement: Supplementary file 1 [file Data_Sheet_1.PDF]

Supplementary Material

1 Supplementary Figures and Tables

1.1 Supplementary Figures

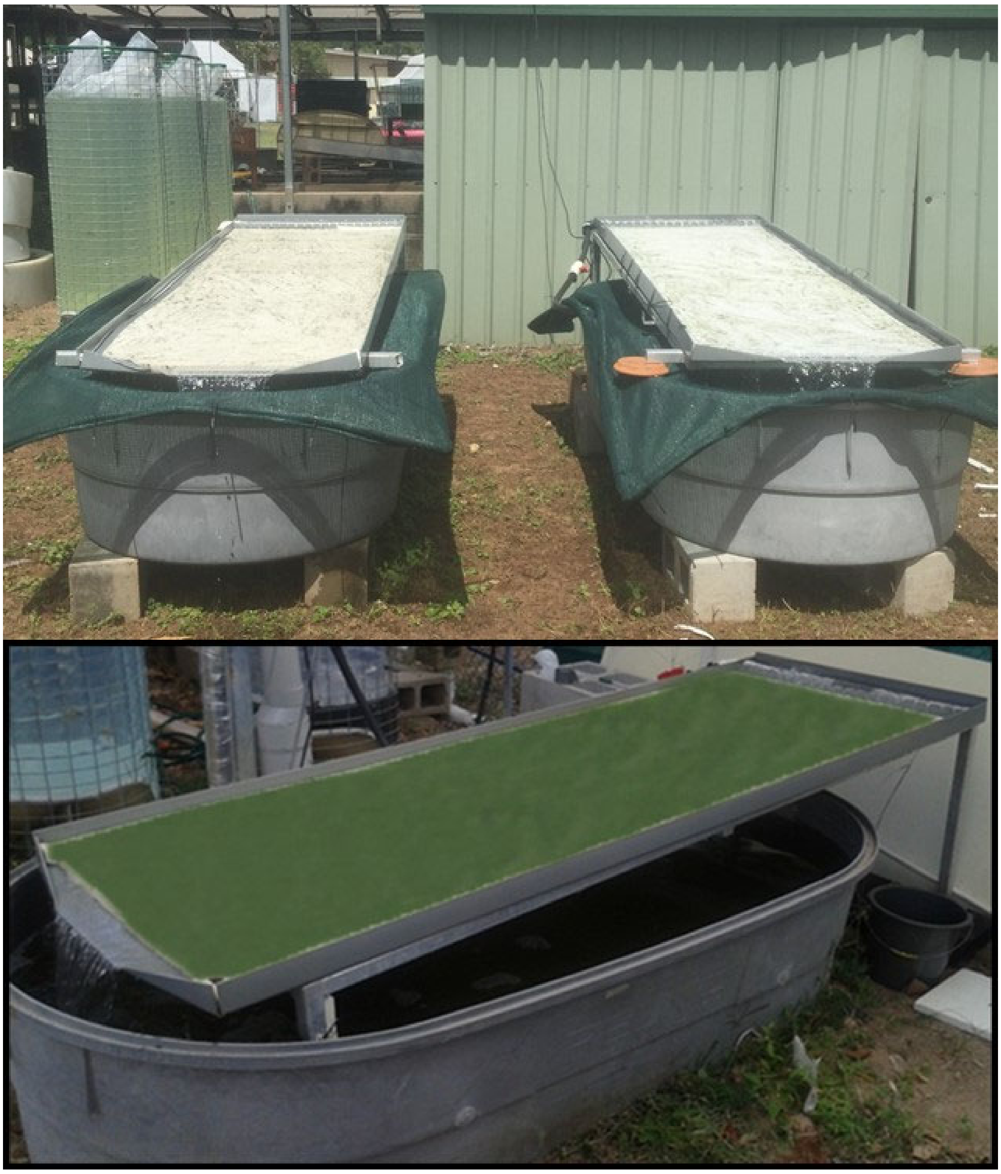

Supplementary Figure S1. Modified algal turf-scrubber used in this study for biofilm cultivation of *Tolypothrix* sp.

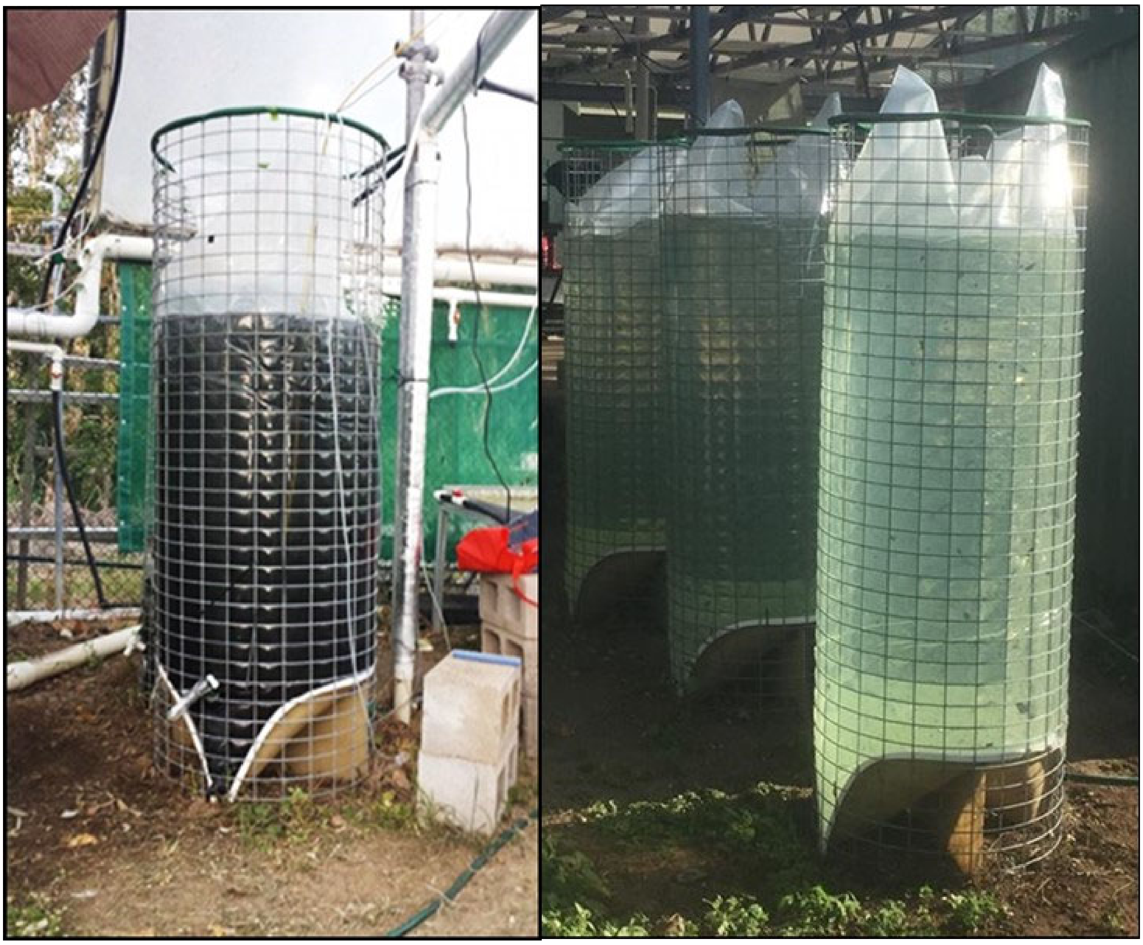

Supplementary Figure S2. Wire cage vertical bag systems used in this study for bubbled suspension culture of *Tolypothrix* sp..

Supplementary Table S1 Characteristics of the algal turf-scrubber (ATS) cultivation system

Parameters	ATS
Cover material	Polystyrene
Area (m^2)	2.2
Polystyrene thickness (cm)	~2.0
Raceway dimensions (length x with) (m)	2.2x1
Tank volume (L)	500
Slope (%)	7
Pump flow (L min^{-1})	66
Flow delivery mode	Continues
Water speed (m s^{-1})	0.6
Water depth (cm)	~0.5
Estimated residence time (slope) (s)	4 (0.8%)
Estimated residence time (tank) (s)	450 (99.2%)

Supplementary Table S2 Characteristics of the vertical bag cultivation system

Parameters	Vertical bag
Wire cage height (m)	1.9
Wire cage diameter (m)	2.0
Vertical bag height (m)	2.0
Vertical bag diameter (m)	1.9
Footprint area (m^2)	0.3
Bag volume (L)	500
Aeration	Continues
Air flow (L min^{-1})	0.05
Tap height (m)	0.1

Supplementary Table S3 Environmental parameters during the study at the freshwater compound, James Cook University, Australia. (Parameters refers to noon)

Culture type	System	Media	Run	Dates	Water temperature (ºC)	pH	Light irradiance (μmol photons m^{-2} s^{-1})
Suspension culture	Vertical bag	SADW	1	September 2016	25 -30	7.0 – 9.0	500-900
		SADW+CO_2			25 -30	6.0 – 9.0	500-900
		SADW	2	October 2016	25-32	7.0 – 9.0	500-900
		SADW+CO_2			25-32	6.0 – 9.0	500-900
Biofilm	ATS	SADW	1	September 2016	25 -30	7.0 – 9.0	500-900
		SADW+CO_2			25 -30	6.5 – 9.0	500-900
		SADW	2	October 2016	25-32	7.0 – 9.0	500-900
		SADW+CO_2			25-32	6.5 – 9.0	500-900
